# Sustainability, spread, and scale in trials using audit and feedback: a theory-informed, secondary analysis of a systematic review

**DOI:** 10.1186/s13012-023-01312-0

**Published:** 2023-10-26

**Authors:** Celia Laur, Zeenat Ladak, Alix Hall, Nathan M. Solbak, Nicole Nathan, Shewit Buzuayne, Janet A. Curran, Rachel C. Shelton, Noah Ivers

**Affiliations:** 1 Women’s College Hospital Institute for Health System Solutions and Virtual Care, 76 Grenville Street, Toronto, ON M5S 1B2 Canada; 2 Institute of Health Policy, Management and Evaluation, Health Sciences Building, University of Toronto, 155 College Street, Suite 425, Toronto, ON M5T 3M6 Canada; 3 Ontario Institute for Studies in Education, University of Toronto, 252 Bloor Street West, Toronto, ON M5S 1V6 Canada; 4 School of Medicine and Public Health, The University of Newcastle, Newcastle, NSW Australia; 5 National Centre of Implementation Science, The University of Newcastle, Newcastle, NSW Australia; 6 Hunter Medical Research Institute, New Lambton Heights, NSW Australia; 7 Hunter New England Population Health, Hunter New England Local Health District, Newcastle, NSW Australia; 8 Physician Learning Program, Continuing Medical Education and Professional Development, Cumming School of Medicine, University of Calgary, 3280 Hospital Drive NW, Calgary, Alberta T2N 4Z6 Canada; 9 Health Quality Programs, Queen’s University, 92 Barrie Street, Kingston, ON K7L 3N6 Canada; 10 School of Nursing, Faculty of Health, Dalhousie University, Halifax, NS B3H 4R2 Canada; 11 Department of Sociomedical Sciences, Mailman School of Public Health, Columbia University, New York, NY USA; 12 Department of Family and Community Medicine, University of Toronto, 500 University Ave, Toronto, M5G 1V7 Canada

**Keywords:** Audit and feedback, Sustainability, Spread, Scale, Implementation, Systematic review

## Abstract

**Background:**

Audit and feedback (A&F) is a widely used implementation strategy to influence health professionals’ behavior that is often tested in implementation trials. This study examines how A&F trials describe sustainability, spread, and scale.

**Methods:**

This is a theory-informed, descriptive, secondary analysis of an update of the Cochrane systematic review of A&F trials, including all trials published since 2011. Keyword searches related to sustainability, spread, and scale were conducted. Trials with at least one keyword, and those identified from a forward citation search, were extracted to examine how they described sustainability, spread, and scale. Results were qualitatively analyzed using the Integrated Sustainability Framework (ISF) and the Framework for Going to Full Scale (FGFS).

**Results:**

From the larger review, *n* = 161 studies met eligibility criteria. Seventy-eight percent (*n* = 126) of trials included at least one keyword on sustainability, and 49% (*n* = 62) of those studies (39% overall) frequently mentioned sustainability based on inclusion of relevant text in multiple sections of the paper. For spread/scale, 62% (*n* = 100) of trials included at least one relevant keyword and 51% (*n* = 51) of those studies (31% overall) frequently mentioned spread/scale. A total of *n* = 38 studies from the forward citation search were included in the qualitative analysis. Although many studies mentioned the need to consider sustainability, there was limited detail on how this was planned, implemented, or assessed. The most frequent sustainability period duration was 12 months. Qualitative results mapped to the ISF, but not all determinants were represented. Strong alignment was found with the FGFS for phases of scale-up and support systems (infrastructure), but not for adoption mechanisms. New spread/scale themes included (1) aligning affordability and scalability; (2) balancing fidelity and scalability; and (3) balancing effect size and scalability.

**Conclusion:**

A&F trials should plan for sustainability, spread, and scale so that if the trial is effective, the benefits can continue. A deeper empirical understanding of the factors impacting A&F sustainability is needed. Scalability planning should go beyond cost and infrastructure to consider other adoption mechanisms, such as leadership, policy, and communication, that may support further scalability.

**Trial registration:**

Registered with Prospero in May 2022. CRD42022332606.

**Supplementary Information:**

The online version contains supplementary material available at 10.1186/s13012-023-01312-0.

Contributions to the literature

• This study explores the understudied area of how sustainability, spread, and scale are discussed in audit and feedback trials.
• The need to consider sustainability is mentioned frequently, but little detail is provided on how to plan for audit and feedback to be sustained, if found to be effective.
• The time periods used to explore sustainability were relatively short. Twelve months was the most frequently mentioned sustainability period.
• When planning for scaling-up, trials most frequently mentioned the need to keep costs low and use existing infrastructure.
• Future audit and feedback trials are encouraged to publish follow-up studies that report on sustainability, spread, and scale.

## Introduction

In 2012, a Cochrane systematic review found that audit and feedback (A&F) can have a small, yet potentially meaningful impact in professional clinical practice [1]. Given this impact, sustainability is important to consider to ensure positive benefits are continued. Efforts to ensure sustainability are also important so research funding is not wasted, and the trust of the community is maintained [2–8]. To extend benefits outside the initial trial context, there is also a need to actively consider how A&F might be applied in other settings and contexts (spread) [9] and across a wider area (scale) [10].

Given the potential for beneficial impact and use at a large scale, such as throughout a geographic region or healthcare system, a deeper understanding of how trial teams plan for the A&F to be continued (if effective) in other settings or contexts is needed. In the past 10 years, there has been an influx of A&F trials and an update of the Cochrane review is underway in 2023 [11]. This update provided an opportunity to explore the understudied areas of sustainability, spread, and scale of A&F trials. Although understanding sustained effectiveness of A&F trials will be crucial, and the subject of future research, including specifying if the A&F strategy or the effect on clinical practice was sustained, given the heterogeneity of definitions of sustainability, spread, and scale, and the lack of a standardized sustainability duration period [2, 3], there is a need to first explore how sustainability, spread, and scale are described in A&F studies, before focusing on effectiveness. As sustainability of beneficial effects could be considered in all studies, yet is not typically the focus of many implementation trials, a broad approach was taken to inform and provide a basis for future work. The objectives of this study were to determine how A&F trials describe and plan for 1) sustainability and 2) spread and scale.

## Methods

### Study design

This is a secondary analysis of a Cochrane systematic review using qualitative synthesis methods informed by relevant theory. The focus was on keywords used to describe the three concepts, the timeframe used to claim the impact or overall intervention, including A&F, was sustained, the determinants of sustainability, and the sequence, mechanisms, and underlying factors for spread and scale.

### Operational definitions and theoretical frameworks

For this review, we used the Moore et al. definition of sustainability that is, *after a defined period of time, a program, clinical intervention, and/or implementation strategies continue to be delivered and/or individual behavior change (i.e., clinician, patient) is maintained; the program and individual behavior change may evolve or adapt while continuing to produce benefits for individuals/systems* [12]. Within A&F trials, sustainability can be viewed as having the A&F continue to be delivered while measuring for continued impact on health or behavioral outcomes of interest, or stopping the A&F delivery and measuring for continued impact. Although trials sometimes refer to A&F as an evidence-based intervention or as an implementation strategy, the term A&F process or strategy is used throughout to distinguish implementation strategies from the clinical interventions that those strategies sought to encourage.

To explore determinants of A&F sustainability, the Integrated Sustainability Framework (ISF) was selected as it is theoretically and empirically informed, and identifies common determinants across key levels and domains that have been found to influence sustainability across a range of types of settings and populations [7]. Key domains in the ISF include outer/policy context, inner/organizational context, implementation processes, provider/implementer characteristics, and characteristics of the intervention [7], with determinants that are important to consider within each of those domains (e.g., staffing turnover, cost).

The terms “spread” and “scale” are often used interchangeably; however, for this work, they are defined separately. Spread is defined as “replicating an initiative somewhere else (i.e. one site to another)” [9]. Scale is defined as “deliberate efforts to increase the impact of successfully tested health innovations so as to benefit more people and to foster policy and program development on a lasting basis” [10]. As included studies are all trials, the number of sites included may be due to study design requirements, rather than purposefully spreading or scaling the A&F process. As there are still important learnings regarding spread/scale from implementing trials at multiple sites, the reason for the number of sites should be kept in mind while interpreting these results. To gain a deeper understanding of factors to consider when planning for scale, the Framework for Going to Full Scale (FGFS) was used, which includes the phases of scale-up, adoption mechanisms, and support structures (infrastructure) [13].

### Search strategy and information sources

The updated Cochrane review includes trials from the previously published version of the review (*n* = 140 originally, with *n* = 117 included in the updated review) [1, 11], as well as (*n* = 170) trials identified from electronic searches of the following databases: Cochrane Central Register of Controlled Trials (CENTRAL), MEDLINE, EMBASE, CINAHL, clinicaltrials.gov, and WHO International Clinical Trials Registry Platform. The initial search was limited to trials published from 2010 to June 2020 (*n* = 121), with an updated search from June 2020 to January 2022 (*n* = 40 additional studies). Details on the search strategy for the Cochrane review are provided in the protocol [11].

### Eligibility criteria

Trials with A&F as the core strategy or as part of a multi-component intervention were considered eligible for the updated review [11]. All trials included in the updated review published between 2011 and January 2022 were included. The 2011 cut-off was selected to align with the seminal paper by Scheirer and Dearing which increased the focus on sustainability considerations in research [2].

### Data screening and extraction process

Data extraction included identification of keywords (yes/no); study duration (months); sustainability period (months, if relevant); author mention of measuring sustainability (yes/no); and the copying of relevant text from the main paper and supplemental files relevant to sustainability, spread, and scale. Location (abstract, introduction, etc.) of relevant text in the main file was included. Extraction was piloted in two rounds by four researchers (CL, ZL, AH, and NS), using feedback from each pilot to refine our strategy.

Duplicate extraction of included studies was completed independently by 6 researchers (CL, ZL, AH, NN, NS, and JC). Sustainability keywords included sustain*, maint*, institutional*, integrat*, normal*, embed*, durabil*, longitudinal*, long*-term, routine*, and standard*. Spread and scale keywords included spread*, scal*, roll* out, reach, and generali#e*. Keywords were initially identified from reviews with relevant search strategies for sustainability [14] and spread/scale [15]. Extractors could list additional relevant words identified. Only keywords within the appropriate meaning were included (i.e., mention of approval from the “institutional” review board would not be included). Negative instances (i.e., no focus on sustainability) were included as our focus was on all mentions of these terms in the context of A&F trials. Discrepancies were decided by CL. A full list of keywords is included in Additional file 1: Full list of keywords.

Extraction only continued for studies with at least one keyword for either search (sustainability or spread/scale), while studies without a keyword were removed. For studies with a keyword, each relevant passage of text was copied along with the location of the text. For sustainability studies, total study duration (including baseline data) was extracted along with duration of the period over which sustainability was assessed, which was qualified as after the intervention period and was referred by trial authors by multiple names (i.e., follow-up, maintenance phase). Studies needed a minimum of three data collection points to qualify as having a sustainability period (i.e., (1) pre-intervention or strategy; (2) post-intervention or strategy; (3) sustainability). Whether or not the author claimed to be measuring sustainability was also extracted as this did not always align with inclusion of a sustainability period based on our definition. For supplemental files, relevant text was copied and included separately. When merging the duplicate coding, all relevant text copied by each extractor was included for analysis.

### Forward citation search

One researcher (CL) conducted a forward citation search between July and December 2022 for each included study following methods suggested by Brown University [16]. Publications which cited the included study were identified through PubMed Central using the “Cited By” feature which produced a list of studies that was screened by title and abstract, followed by full text review of relevant studies. Studies that directly connected to the original study and considered sustainability or spread/scale were included. For example, a brief report publishing the 12-month results after a 6-month study would be included, or a study that applied the same intervention, including A&F, in a new setting. Forward citation studies were not included in the keyword search; however, text related to sustainability, spread, and scale was extracted.

### Data analysis

Results from the keyword searches were analyzed descriptively, along with sustainability phase durations, and information on whether the authors claim to be measuring sustainability. Descriptive results per trial (year of publication etc.) are based on extraction from the wider updated Cochrane review (in press).

Due to the variation in the amount of focus each study placed on sustainability and spread/scale, there was a need to group studies prior to analysis. Based on pilot data extraction and analysis of 15 studies, we differentiated between “frequent” and “occasional” mentions of relevant text. Frequent sustainability includes all studies that had sustainability-related text extracted from three or more locations (abstract, introduction etc.). Occasional sustainability includes all studies that had sustainability-related text extracted from one to two locations. Frequent spread/scale includes all studies that had spread/scale-related text extracted from two or more locations. Occasional spread/scale includes all studies that had spread/scale-related text extracted from one location.

Studies defined as “frequent” underwent comprehensive inductive content analysis and deductive analysis to the ISF or FGFS. Studies with “occasional” mentions underwent content analysis only and were not mapped to a framework. As the keyword “generalizabl*” was deemed to have a relevant but unique meaning, studies that were only included because of this keyword were grouped separately. See Additional file 2: Methods for grouping studies.

All qualitative analysis was conducted by two researchers (CL and ZL) using NVivo 12.

Piloting of the codebook (Additional file 3: Codebook) was conducted by CL and ZL for five studies each in frequent sustainability and frequent spread/scale. The codebook for frequent sustainability was based on definitions adapted from Shoesmith et al., which were designed with the original developers of the ISF [17]. The codebook for frequent spread/scale was based on the FGFS descriptions provided by Barker et al. [13].

As no differences in the content analysis were found between studies with the occasional sustainability and spread/scale groupings, results were merged with the frequent groupings. Text extracted from supplemental files (protocols, theses, appendices etc.) and the forward citation search was analyzed by one coder (CL).

## Results

There were 161 included studies. Thirty percent (*n* = 49) were published in the USA, 85% (*n* = 137) were parallel cluster randomized control trials (RCTs), and 46% (*n* = 74) were conducted in a primary care setting (Table 1).

**Table 1:** Summary of trial descriptives for all studies and separated by sustainability and spread/scale groupings

	**All studies** ***n*** ** (%)**	**Frequent sustainability** ***n*** ** (%)**	**Occasional sustainability** ***n*** ** (%)**	**Frequent spread/scale** ***n*** ** (%)**	**Occasional spread/scale** ***n*** ** (%)**
**Total # studies**	161	62	64	51	14
**Country**					
USA	49 (30%)	15 (24%)	24 (38%)	15 (29%)	3 (21%)
Europe	40 (25%)	14 (23%)	15 (23%)	5 (10%)	2 (14%)
Canada	21 (13%)	8 (13%)	9 (14%)	7 (14%)	4 (29%)
Australasia	16 (10%)	8 (13%)	6 (9%)	8 (16%)	2 (14%)
Asia	11 (7%)	5 (8%)	3 (5%)	3 (6%)	1 (7%)
UK	9 (6%)	7 (11%)	2 (3%)	7 (14%)	0
Africa	7 (4%)	4 (6%)	3 (5%)	4 (8%)	1 (7%)
South America	4 (3%)	0	1 (2%)	2 (4%)	1 (7%)
Middle East	2 (1%)	0	1 (2%)	0	0
Multi-region	2 (1%)	1 (2%)	0	0	0
**Design**					
Parallel cluster RCT	137 (85%)	50 (81%)	55 (86%)	46 (90%)	11 (79%)
Step wedge	23 (14%)	11 (18%)	9 (14%)	5 (10%)	3 (21%)
Cluster randomized crossover	1 (1%)	1 (2%)	0	0	0
**Setting**					
Primary care	74 (46%)	35 (56%)	22 (34%)	26 (51%)	8 (57%)
Hospital inpatient	45 (28%)	14 (23%)	23 (36%)	11 (22%)	4 (29%)
Other outpatient clinic	16 (10%)	7 (11%)	6 (9%)	4 (8%)	0
Community care	9 (6%)	4 (6%)	3 (5%)	5 (10%)	1 (7%)
Emergency departments	4 (2%)	1 (2%)	2 (3%)	1 (2%)	0
Mixed	7 (4%)	0	5 (8%)	1 (2%)	1 (7%)
Other	6 (4%)	1 (2%)	3 (5%)	3 (6%)	0

*RCT* randomized control trial

For sustainability, within the 78% (*n* = 126) of studies with at least one keyword, 49% (*n* = 62; 39% overall) qualified as frequent sustainability. For trials grouped as occasional sustainability, 28% (*n* = 35/126; 22% overall) had text in two locations, and 23% (*n* = 29/127; 23% overall) with text in only one location. For spread/scale, within the 62% (*n* = 100) of studies with at least one keyword, 51% (*n* = 51/100; 32% overall) qualified as frequent spread/scale. For trials grouped as occasional spread/scale, 14% (*n* = 14/100; 9% overall) had text in one location. Thirty-five percent (*n* = 35/100; 22% overall) of trials only mentioned generalizability.

The forward citation search yielded *n* = 2698 studies; *n* = 122 for title/abstract review, *n* = 46 for full text review, for a total of *n* = 38 included. For sustainability, *n* = 28 new studies were included and linked to *n* = 19 original studies (*n* = 15 frequent sustainability). For spread/scale, *n* = 18 new studies were linked to *n* = 12 original studies (*n* = 7 frequent spread/scale; *n* = 3 generalizability only). Supplemental files were included for sustainability studies (*n* = 18) and spread/scale studies (*n* = 14). No new themes were identified from the supplemental files and extracted text was merged with the overall results. Although forward citation studies provided valuable information on sustained results, application of implementation theories, and protocols for future studies to sustain or scale-up the original results, no new themes were identified.

**Fig. 1:**
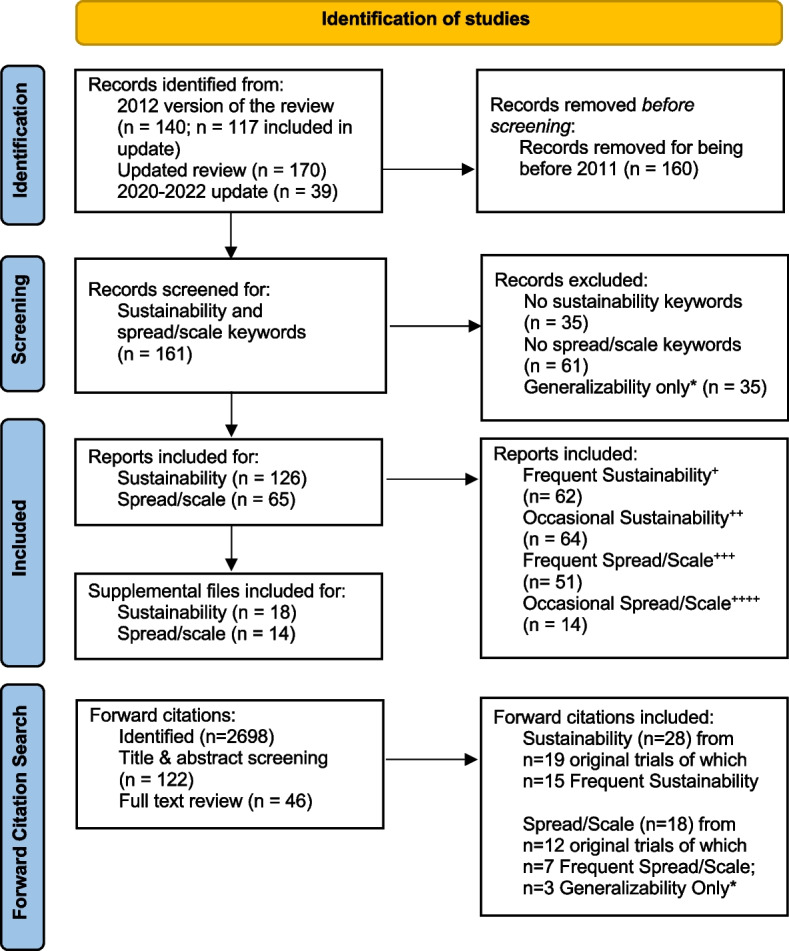
PRISMA statement of included and excluded studies separated by sustainability and spread/scale. *Generalizability only refers to studies that were only included for mentioning the term “generalizability” and were therefore removed. ^+^Frequent sustainability includes all studies that had sustainability-related text extracted from three or more locations (abstract, introduction etc.). ^++^Occasional sustainability includes all studies that had sustainability-related text extracted from 1 to 2 locations (abstract, introduction etc.). ^+++^Frequent spread/scale includes all studies that had spread/scale-related text extracted from two or more locations (abstract, introduction etc.). ^++++^Occasional spread/scale includes all studies that had spread/scale-related text extracted from one location (abstract, introduction etc.)

**Fig. 2:**
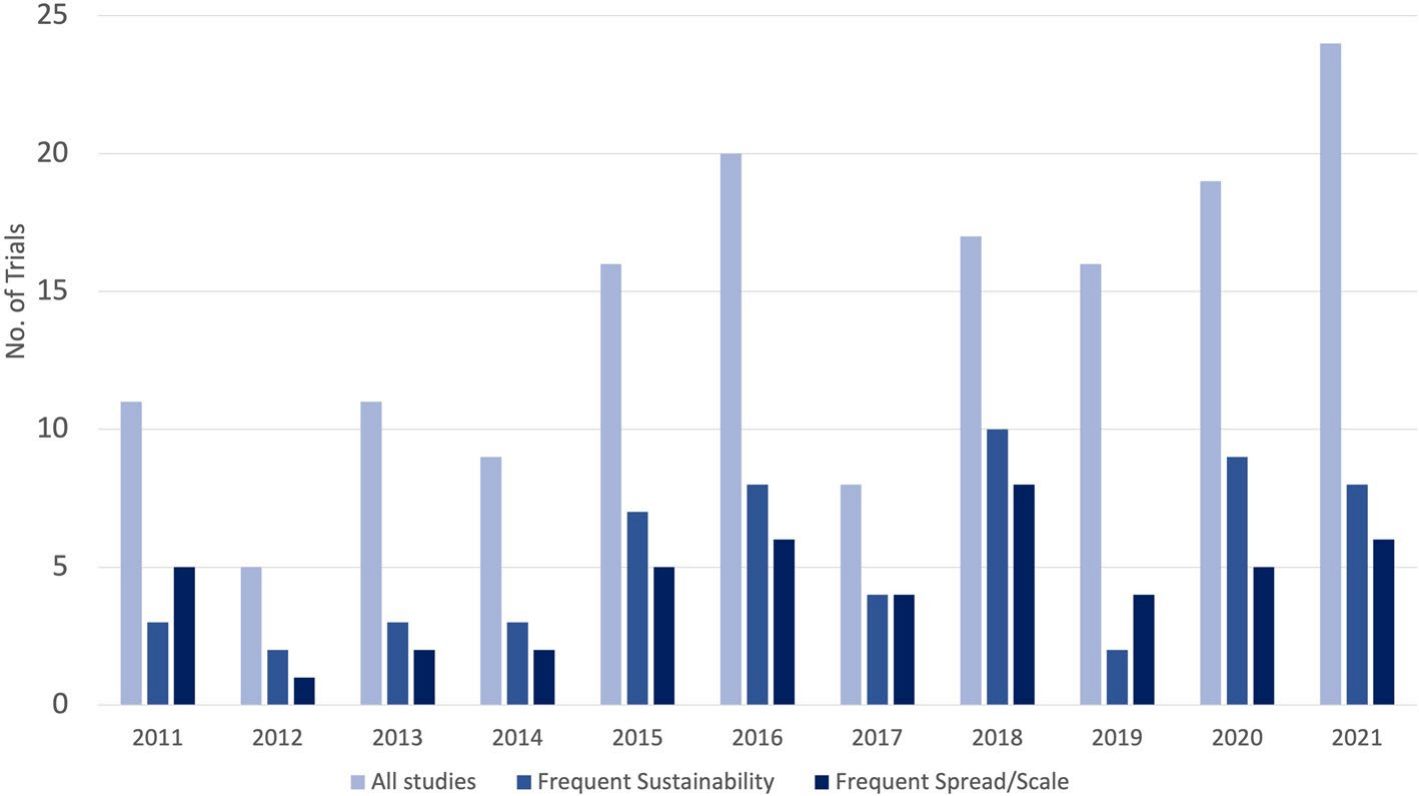
Summary of publication year for all trials, and those with frequent mentions of sustainability, and spread/spread. (2022 is excluded as only January data is available.)

A summary of study inclusion is provided in Fig. 1. Descriptives of the trials are provided by groupings (Table 1) and by year of publication (Fig. 2). Figure 2 shows no trend regarding the number of keywords found for sustainability, spread, or scale over the past 10 years.

Extracted text for sustainability fit within the broader ISF determinants (organizational context etc.); however, lack of details specific to A&F made it difficult to identify determinants (barriers and facilitators) directly impacting sustainability. For spread/scale, strong alignment was found with the FGFS for phases of scale-up, and support systems (infrastructure), but not for adoption mechanisms. Three new themes were identified including aligning affordability and scalability; balancing fidelity and scalability; and balancing effect size and scalability.

### Keywords

**Fig. 3:**
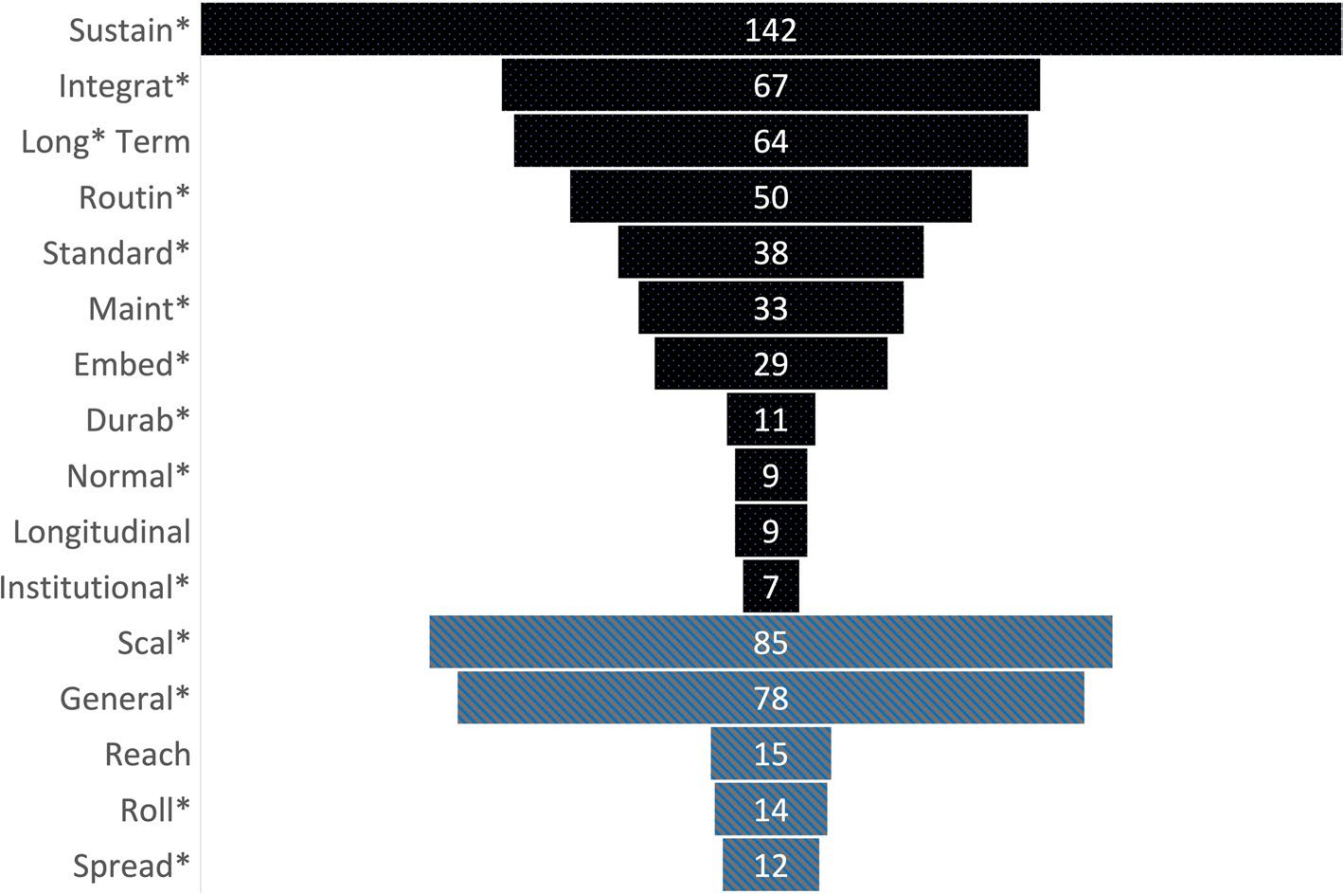
Keyword counts for sustainability and spread/scale across all studies (*n* = 161). This count includes multiple keywords per study. The dark/black bars represent the sustainability keywords, and the lighter/gray bars represent the spread/scale keywords. *Word stem. Full list of words is provided in Additional file 1: Appendix 1

For sustainability, the most frequent keyword mentioned was “sustain*” (*n* = 142), followed by “integrat*” (*n* = 67) and “long*-term” (*n* = 64). For spread/scale, the most frequent was “scal*” (*n* = 85), with only *n* = 12 mentions of “spread.” Word counts include negative instances, such as when studies did not measure sustainability. The full keyword count is included in Fig. 3.

### Sustainability

#### Trial durations

The total duration of all trials that included at least one keyword regarding sustainability (*n* = 126), ranged from 2 to 75 months, for an average of 21 months, with 24 months being the most frequent total duration. Of those with a sustainability period mentioned (*n* = 37 based on our definition), duration ranged from 2 to 24 months, for an average of 10.4 months. Multiple study types were included. Twelve months was the most frequent sustainability duration. Although *n* = 37 trials claimed to measure sustainability, two of the studies did not report a timeframe. Two separate studies did not claim to measure sustainability, but had at least two time points measured after the intervention period, which may be due to a need for multiple time points for analysis rather than a focus on sustainability.

#### Key themes

Most studies that mentioned sustainability indicated they needed a longer trial duration and/or that more research was needed to determine sustainability of their overall intervention, which would include A&F. In several studies, there were inconsistencies in how studies reported whether or not results were sustained. Explanations of sustained effect were typically predictions or interpretations in the discussion, rather than direct results, such as from a process evaluation. Most studies indicated the overall intervention, including A&F, stopped after the trial ended, some continued, and others did not mention either way. Some trials determined the need for ongoing A&F, while others thought occasional “booster” sessions could encourage sustained change. Multi-component interventions rarely discussed sustainability determinants for individual components of the intervention, and typically provided more generic statements.

#### Integrated Sustainability Framework

Determinants of the ISF were used for deductive analysis. Determinant descriptions, ISF factors, and supporting quotes are provided in Table 2. Not all determinants described within the ISF were identified.

**Table 2:** Domains and determinants adapted from the Integrated Sustainability Framework (ISF), along with key quotes from included audit and feedback trials

Domains and determinants	Quotes
**Outer/policy context** The external landscape, including policies, regulations, and guidelines. The availability of funding to maintain the intervention, the role of external partnerships, broader environmental support, and alignment with broader values, priorities, and needs. *ISF determinants:* • Policy and legislation • Sociopolitical context • Funding environment • Leadership • Values, priorities, needs • Community ownership	*Since 2009, China has enacted national health policy reforms to regulate antibiotic prescribing. … Our recent study showed that, at the county hospital level, the policy might be associated with reducing inappropriate antibiotic prescribing in outpatients.* [18] *Limitations include our focus on commercially insured patients within a single integrated delivery system that had the ability to mobilize resources even in the absence of external funding. Systems that are smaller, are located in other geographic regions, or serve primarily publicly insured patients may have fewer resources or may face different challenges in reaching vaccine providers.* [19] *Hospitals were expected to implement the national perioperative safety guidelines. However, it is not easy to implement new guidelines and sustain change.* [20] *Several structural and environmental barriers for implementing evidence-based practices within LTC* [Long Term Care] *homes have been identified including a high proportion of unregulated staff, absence of a learning culture, high turnover in management, heavy regulatory and documentation demands, routinized care rituals, and lack of familiarity with clinical practice guidelines.* [21]
**Inner/organizational context** The impact of the organizational structure, leadership/support, readiness of change, resources available, and organizational stability, including staff turnover. *ISF determinants:* • Funding/resources • Leadership/support • Climate/culture • Staffing turnover • Structural characteristics • Capacity • Champion • Polices (alignment) • Mission	*The implementation packages were tested under ‘real-world’ conditions, increasing confidence in wider applicability to routine general practice settings.* [22] *Adequate infrastructure such as information and communications technology was often lacking.* [20] *Consideration should be given to the intervention ‘fit’ with existing systems and staff skills, and patient groups, including how best to facilitate local tailoring and embed the intervention within routine care.* [23] *It is possible that staff turnover led to loss of ‘corporate memory’ about chlamydia, contributing to reduced testing.* [24] *The findings illustrate that there may be different factors at play during initial implementation compared to those that are needed to influence sustained use of the intervention. There appear to be spheres of influence that when aligned enhance normalisation of the intervention into routine practice. The first broadly relates to the mission of the site, its organisational culture and the antecedents to participating in this project. The second related to the leadership structures and the role of influential leaders in changing the activities of others. Third relates to the team environment and the extent to which certain actors within the team influence the activity of others. The fourth relates to the tools themselves and the degree to which they are fit-for-purpose from content, workflow and technical perspectives.* [25] forward citation from [26]
**Implementation processes** Description of how the intervention is implemented, including the role of key decision makers, the training and support provided to the implementation team, the mechanisms for evaluating the program and collecting data, if, and how, the program can be adapted to meet the continually changing needs of the patients and organization, and the strategic planning for the future of the intervention. *ISF determinants:* • Partnership/engagement • Training/support/ supervision • Fidelity • Adaptation • Planning • Team/board functioning • Program evaluation/data • Communication • Technical assistance • Capacity building • Implementation science* (new)	*By integrating this intervention into routine care and making all material freely available at the end of the intervention, the* [name] *study strives to be sustainable and self-promoting and, thereby, implemented in primary care in Ireland beyond the intervention period.* [27] protocol of [28] *The tool components were synergistically incorporated into the practice with the manager taking ownership of the audit tool and the GP focusing on the in-consultation decision support tool. This facilitated initial adoption of the intervention; however, sustained engagement of the research team was required suggesting a lack of normalisation beyond the trial setting.* [25] forward citation from [26] *The implementation packages embedded behaviour change techniques within audit and feedback, educational outreach and (for risky prescribing) computerised prompts.* [29] *We set out to design and apply an implementation package that could be delivered sustainably using resources typically available to primary care. We involved health professionals, commissioners and patients in structured deliberations to prioritise and develop a set of ‘high-impact’, evidence-based Qis associated with scope for improvement and that could be measured using routinely collected data.* [29] *The pragmatic optimization approach featured in this aim was designed in close partnership with our research collaborators to model the considerations healthcare decision-makers told us they actually use when making decisions about adopting and sustaining evidence-based practices.* [30] forward citation of [31] *Tailored interventions appeared to lead to more sustainable compliance increases.* [32] *Each practice was allowed to consider how to best integrate the referrals into their workflow, allowing variation in implementation fidelity.* [33]
**Provider/implementer characteristics** Specific provider and implementer characteristics, such as roles, motivations, attitudes, benefits, stressors, skills, and expertise. *ISF determinants:* • Provider/implementer characteristics • Implementation skills/expertise • Implementer attitudes • Implementer motivation • Population characteristics (removed)	*Many participants were insufficiently motivated to change established behaviour patterns and procedures.* [34] *The formation and maintenance of site-based quality improvement teams that aimed to lead local barrier identification, solution generation, solution implementation, and goal setting were notable deficiencies at many intervention sites.* [29] *When discussing the indicators and associated clinical behaviours, primary care professionals generally viewed the workload and burden associated with adherence as accepted and embedded components of general practice.* [21] *Participants considered that researchers did not have a good understanding of the way general practice operates, suggesting a number of reasons why the research might be difficult to sustain within the general practice environment.* [35]
**Characteristics of the intervention** How much the intervention can be adapted, how it fits within the context, population or organization, the perceived benefits or impact of the intervention and the need for this benefit within the community or setting where it is being implemented. The burden and complexity of the intervention is also covered as well as the cost. *ISF determinants:* • Adaptability • Fit with population and context • Benefits/need • Burden/complexity • Trialability • Cost	*Hospitals are complex dynamic systems, and shifting behavior may take longer than expected. Despite multiple modalities targeting system and individual factors in an active and interactive way, it was only in the past 4 months of the 16-month intervention period that a shift in implementation was evident.* [36] *The* [name] *intervention is feasible in primary care and preliminary results suggest a positive impact on uptake. However, consideration should be given to the intervention ‘fit’ with existing systems and staff skills, and patient groups, including how best to facilitate local tailoring and embed the intervention within routine care.* [23] *While most staff (86%, n* = *19) agreed the intervention was doable, only 71% (n* = *15) agreed it was easy to use…. Intervention delivery was feasible during the study period, but the intervention was an ‘extra thing’, and there were mixed views on the sustainability of specific components.* [23] *Because multilevel interventions require substantial investments of personnel and time in the short-term, demonstrating that intervention effects continue in the post intervention* *period is important when clinical and policy decision makers consider upfront costs.* [37] *There is a high-cost barrier for one-off audit and feedback interventions.* [38] *This is consistent with evidence that adherence to clinical recommendations that are more complex or disruptive to routine practice is lower compared with simpler recommendations.* [22]

#### Outer/policy context

The ISF determinant of outer/policy context represents the impact of the external landscape (policies, funding availability, partnerships, fit with national values etc.) on sustainability. There was minimal mention of how this external context impacted A&F trials. When mentioned, focus was on implementing new guidelines, and how external partners facilitate long-term implementation. One study saw potential for “*embedment in a national quality assurance cycle*” [39] to support sustainability. Access to external funding was a barrier, yet the focus was on the cost of the intervention rather than the broader funding landscape. Any mention of alignment with national or regional values was about the need to consider these values, not how they should be considered, as shown by this study: “*We would suggest this includes due attention to influencing the institutional culture and context of rural hospitals although willingness to invest in more integrated approaches often seems lacking*” [35].

#### Inner/organizational context

Inner/organizational context represent the impact of the organizational structure, leadership, and support, as well as readiness to change, access to resources, and organizational stability, including staff turnover. Some trials designed their interventions for “real-world” conditions, with the intent to be sustainable. “*Interventions need to fit with the ‘bigger picture’ of the organisation*” [23]. Access to existing organizational infrastructure was mentioned in plans for long-term implementation and was predicted to impact future sustainability; however, this was rarely actioned or followed up with empirical data, with most studies only providing the recommendation. Access to an electronic medical record (EMR) to generate local data, the need to involve local staff, and access to existing resources were all suggested to impact sustained integration into the organization. “*Translation of the trial results is readily feasible because the interventions are delivered using the practice systems that are employed in delivering routine care*” [34].

There were many concerns about an organization’s ability to keep trials going long-term. “*Although managers were pleased with the improvements in prescribing performance, they were in agreement that the intervention program was too labour- and resource-intensive for long-term implementation*” [40]. Concerns included lack of supportive infrastructure or an organization’s ability to continue without researchers. “*Many hospitals lack the resources or expertise to organise and lead an implementation effort or to manage the changes needed, collect data, and initiate improvement teams*” [20].

#### Implementation processes

Implementation processes consider how the intervention is implemented (decision maker involvement, implementation team training and support, program evaluation, adaptation, strategic planning etc.). Within trials that planned for sustainability, focus was on how to embed the intervention into routine practice. This embedding was thought to be supported by involvement of key decision makers and local staff, mainly in the design process, and connected to ongoing adaptation. “*Our* [intervention] *consisted of comparable standardized elements, but more strongly involved local professionals in the design and performance of the locally tailored interventions*” [41]. The ability to tailor the intervention (including A&F) to changing patient and organizational processes was said to support embedding, but mainly how to tailor in the future, as changes were not typically made during the trial. “*The stepped-wedge design did not allow us to anticipate in a flexible manner to all types of circumstances that hindered the implementation. In retrospect, it is fair to say that we expected too much change in a too short time frame*” [20]. In studies that did include tailoring, the ability to adapt was generally reported as a facilitator to sustainability. “*Allowing participants to develop tailored systems changes to address barriers may have promoted sustainability by building engagement and aligning efforts with existing clinical processes*” [37].

There was little mention regarding team training for A&F. Strategic planning typically focused on recommendations for what should happen next for effective interventions (including, but not limited to A&F), rather than experience with strategic planning. Program evaluation and access to data focused on the infrastructure for access to audit data, not on data to evaluate the ongoing impact of the A&F strategy.

A new factor was the use of implementation theories, models, and frameworks, and behavior change theory, to strengthen the implementation process and support sustainability potential. “*The principal strength of the study is that it met the requirements of systematic reviews calling for large well-designed long-term trials of hand-hygiene interventions which apply behavioural theory to intervention design*” [42].

#### Provider/implementer characteristics

Specific provider or implementer characteristics, such as roles, benefits, stressors, skills, and expertise, were rarely mentioned. When characteristics were discussed, focus was on embedding with existing staffing models and capacity, as well as motivation of implementers, including champions, to stay involved. Aligning with organizational capacity, the reliance on existing staff was suggested to be beneficial when planning for real-world implementation. “*Using existing staff is important for understanding whether a model is feasible and sustainable regardless of externally funded interventionists*” [43]. Other studies found that what they were asking of local staff was infeasible. “*It appeared that large-scale uptake of evidence-based but complex implementation strategies with a minimum of influence of external researchers, but with the stakeholders in healthcare themselves being responsible for the work that comes with integrating this intervention into their own groups, was not feasible*” [44].

Motivation to stay involved was described as a barrier and a facilitator to sustainability. If there were multiple delays in the implementation process, and lack of time, these decreased initial implementation effectiveness and sustainability potential. “*The operational delays in preparing the Dashboard in the latter months left supervisors with less time to perform their duties and may have reduced the quality of supervision. Second, supervisors could have lost motivation over time, which might have reduced the effectiveness of their supervision*” [45]. Motivation could also be beneficial if implementers, particularly supervisors or champions, maintained enthusiasm and continued to apply and promote the changes. “*An enthusiastic motivator who used her or his time and energy to provide feedback, encourage competition and energize the staff to keep up the efforts throughout the season*” [46].

Population characteristics are typically included in this ISF domain; however, this information would not have been extracted from trials, so it was removed.

#### Characteristics of the intervention

Characteristics of the intervention include the ability of the intervention, including A&F, to be adapted (not how it is adapted), fit within the context, perceived benefit, need for this benefit, burden and complexity of the intervention, and the cost. The A&F trials focused on challenges of working with complex interventions and systems. “*Delivering a complex intervention into a complex system, … is challenging with many barriers to achieving intended outcomes. There was no simple reality*” [20].

Cost was mentioned as a key characteristic impacting sustainability, including comparison between research costs and sustained implementation. “*Although the added costs of such resource-intensive support can be maintained during research evaluations, it is challenging to incorporate these costs into a business model that enables sustainable, scalable provision of the service*” [47].

The fit with the context, population, or organization, as well as the need for the intervention, was mainly covered in the descriptions of the need for the trial itself, not connected to sustainability. Perceived benefits were mainly covered in the results regarding whether or not the intervention, including A&F, was effective, only speculating on the potential for sustained benefit in the discussion.

### Spread and scale

#### Key themes

Most studies made generic statements regarding the need for more studies to consider scale for their specific clinical area and more generally. Within studies that mentioned conducting the trial at scale, many were reported as “first of their kind” and provided some strategies for how they planned for scalability. Strategies were mainly focused on keeping costs low and using existing infrastructure. Many of these same trials recommended that more preparation work was needed and provided suggestions on why the intervention did or did not have the desired effect at scale.

#### Framework for Going to Full Scale

Results of the deductive analysis to the FGFS, specific themes related to A&F, definitions of the FGFS determinants, and supporting quotes are included in Table 3. Additional themes and supporting quotes are provided in Table 4.

**Table 3:** Results from the deductive analysis for spread/scale text to the Framework for Going to Full Scale

Framework definition^a^	Theme	Key quotes
**Phase of scale-up: what phase of the scale-up process is the trial working at?**		
** Phase 1: set-up** Prepares the ground for introduction and testing of the intervention that will be taken to full scale. Establishes an entry point for the planned intervention into the existing health system. Includes a clear articulation of what needs to be scaled up and defines the ambition for “full scale.” Initial test sites, early adopters, and potential “champions” of the intervention are identified.	Materials and training are designed with scalability in mind. Acknowledgment that some tailoring may be required to meet site-specific needs. More set-up/planning/pre-testing needed before scaling up (connects to phase 3).	*We purposely designed this intervention to be relatively low in intensity but wider in reach, maximizing generalizability and dissemination possibilities.* [48] *The intervention in which prescribers received patient leaflets and clinic posters as well as the interactive workshops is low cost to scale-up but it did require intensive development and pretesting with end users.* [49] *We ensured it was as comparable and structured as possible while also allowing site-specific tailoring to address differing clinic needs and to allow a sense of ownership of the project by the healthcare providers implementing the activities.* [50] *We would now recommend more intensive field work involving iterative cycles of testing and refining interventions prior to scaling up for definitive evaluation.* [22]
** Phase 2: develop the scalable unit** An early test and demonstration phase. Scalable unit: typically, a small administrative unit (e.g., sub-district/district or clinical ward/division) that includes key infrastructural components and relationship architecture that are likely to be encountered in the system at full scale. If the ambition of scale is large (e.g., county, province, health system), a scalable unit could comprise multiple levels of care and the communities that are served by a large health system, or a divisional unit of care in a hospital setting or large clinic system.	Goes beyond the design phase by conducting small pilots to understand the intervention and potential for scale.	*To mitigate this risk* [that the trial was perceived as intrusive and disruptive to workflow]*, the solution was trialed in 3 sequential small-scale pilots.* [51] *Before beginning the QI project, 1 author … piloted the training in 1 clinic. He refined the approach on the basis of physicians’ feedback and then prepared physician training leaders to deliver it through an in person “train the trainer” session.* [19] *This eCRT showed that it was feasible to use the* [clinical intervention] *to evaluate interventions that may be readily scaled up to the population level. Feedback received in the eCRT process evaluation, together with evidence from other trials cited above, identifies ways to increase engagement in the intervention and increase effect sizes.* [34] *By first testing different forms of nudges, we could optimize the design of the intervention before implementing and scaling it within the EHR, which involves the additional time and expense of programming new functionality.* [52]
** Phase 3: test of scale-up** Testing the set of interventions to be taken to scale. Spreads the intervention to a variety of settings that are likely to represent contexts that will be encountered at full scale. The underlying theory of change and the change package from a successful early demonstration need to be tested in a broader range of settings before going to full scale. Test necessary infrastructure (e.g., data systems and supply chain) required to support full-scale implementation and build the human capacity and capability (e.g., leadership, managerial, and frontline capacity needed to support the method being used to scale up). Important opportunity to build the belief and will of leaders and frontline staff to support the changes.	Trials conducted before going to full scale. These are larger studies than just pilots (phase 2), as they had multiple sites, settings etc. and aim to be conducted in “real world” conditions. Some differences between sites were found; some allowed for adaptation between sites. (Discussion about infrastructure included below)	*We investigated in a nationwide trial the feasibility and effectiveness of a large-scale, quarterly prescription feedback intervention on antibiotic use in primary care over 2 years using routinely collected claims data in Switzerland.* [53] *We wanted to build on the experiences from the work by Verstappen *et al*. and undertake a large-scale implementation of the strategy in a pragmatic trial with much room for the LQICs to adapt the strategy to their own needs and without any researchers being present embedded within the existing network of LQICs under real-world conditions, increasing confidence in wider applicability to routine general practice settings.* [44] *We sought to evaluate the efforts of a large pediatric health care system to improve HPV vaccination coverage among adolescent patients using existing, research-based materials that were adapted to reflect local stakeholders and settings. … Understanding how large health care systems conduct HPV vaccination QI is important given the potential for system-wide efforts to influence many clinics, providers, and patients.* [19] *This highly pragmatic trial showed the effectiveness of a low intensity feedback intervention delivered by the NHS and implemented across nearly all practices in three geographical areas. With the rapid growth of patient level datasets based on electronic medical records or pharmacy claims data, the potential for feedback interventions to improve prescribing safety is considerable, and many healthcare systems could deploy similar interventions now.* [54]
** Phase 4: go to full scale** Unfolds rapidly to enable a larger number of sites to adopt and/or replicate the intervention. A well-tested set of interventions, supported by a reliable data feedback system, is adopted by frontline staff on a larger scale. The focus is on rapid uptake of the intervention through replication.	Intervention is delivered at scale (population-level, full health system, across a province or country, etc.).	*After a careful scaling of the intervention, ample communication, and stakeholder support, we were able to perform a large-scale randomized controlled trial covering all Australian states.* [51] *Our study shows that quarterly provided prescription feedback over 2 years is possible at low costs on a nationwide scale.* [53] *Our large-scale nationwide study results extend those of a recent single centre study showing ADR improvement with a short educational intervention.* [55] *A population-wide, randomised, intervention trial of audit and feedback to more than 1400 community pharmacies.* [47]
**Adoption mechanisms**		
** Better ideas** Ideas that are designed for scalability. Evident superiority of the intervention. Simplicity. Alignment with the culture of the new implementers.	Focused on the initial ideas/principals used to inform the trial design. Building or tailoring based on the literature.	*We conducted a rapid systematic review to put the results in context, specifically focusing on large, countrywide approaches not involving elements that would be difficult to be implemented on a large scale (such as on-site visits or educational elements).* [53] *We tailored the intervention to conform to principles identified in the literature as associated with improvements in processes and outcomes of care in the ICU: 1) an effective intervention in the ICU must be multifaceted, incorporating education, protocols, and feedback directed at multiple levels of providers … 3) the intervention must be in a format that can be exportable and generalizable to other institutions.* [56] *By first testing different forms of nudges, we could optimize the design of the intervention before implementing and scaling it within the EHR, which involves the additional time and expense of programming new functionality.* [52]
** Leadership** The capacity for leading large-scale change needs to be developed as part of the scale-up process. Leaders can be coached to understand the difference between simply raising awareness of a new practice and what it takes to lead and ensure its broad adoption.	Not found	N/A
** Communication** Communicating the value of the intervention to both leadership and the implementers (frontline staff).	Not found	N/A
** Policy** The identification and/or development of regulatory or administrative policies. Policy can have a supportive or disruptive effect.	Not found	N/A
** Culture of urgency and persistence** Consideration of why others would want to join the effort and whether there is a glaring gap in performance or an urgent need. Checking the amount of will and energy needed to stay the course in bringing interventions to—and achieving results at—full scale. Levels of will and energy may fluctuate over time.	Only focused on urgency about the need for the intervention, rarely about the impact of this urgency.	*That prescribers changed their practice so quickly, and to the extent of almost eliminating use of antimalarial drugs for non-malarial cases in the intervention arms can be interpreted in the context of an increasing national drive for parasite-based malaria diagnosis, with a country-wide scale-up of RDTs that has been ongoing since 2010, which could have raised awareness and readiness for change.* [49]
**Support systems (infrastructure)**		
** Human capability for scale-up** Scale-up will require team leaders who can use change management approaches to guide and mentor teams at the front line and improvement specialists who can lead and design QI-based programs for those who need additional training. The project needs be able to communicate quantitative results and the underlying stories of success and challenge. Data managers need training in analytic and reporting capabilities that are best suited to QI methods (e.g., run charts and statistical process control).	Focus on implementation in “usual circumstances,” including needing minimal implementation support, and trying not to be labor intensive. Less focus on specific skills of team leaders, data managers etc.	*Comprehensive, whole-office-focused interventions are more time-intensive to implement on a large scale, and may involve contributions from non-revenue generating staff (e.g., administrative staff, ADHD care coordinators).* [57] *The implementation of* [study name] *was challenging with the restrictions on logistics, time, and funding, especially when dealing with an intervention requiring behavioural changes and implementation in complex healthcare systems.* [20] *We developed a team-based implementation and engagement model using both a physician expert and a practice facilitator because it quickly became clear that assigning sole responsibility to the physician expert for advising, communicating, and coordinating with change teams (at the clinic) was overly burdensome and not scalable.* [30] forward citation from [31]
** Infrastructure for scale-up** Common structural considerations include: - Additional tools (e.g., checklists, data capture systems) - Communication systems (e.g., materials and messages, mentoring relationships, structured programs) - Key personnel (e.g., data capturers, quality improvement mentors)	Focuses on embedding into existing infrastructure (EHR, existing resources, local talent etc.) to support scale-up. Helpful to scale in systems where organizations use the same system (same EMR etc.).	*Our intervention was a low-cost mechanism, built on existing infrastructure.* [38] *System, structural, and organisational support for system-wide changes to enable implementation strategies to be rolled out and scaled up (e.g., legislation, resources, mechanisms for communication and collaboration between health sectors).* [58] *Given that the national infrastructure needed to support program implementation already exists, widespread dissemination of a modified* [name] *program represents a unique opportunity to address geographic disparities in adolescent vaccination as well as the lackluster uptake of HPV vaccine nationally.* [59] *Our experience suggests that adapting existing materials and harnessing local talent (in the form of physicians who are already high performers) are feasible in the context of a large pediatric health care system and should be considered by other systems as a way to extend reach*. [19]
** Data collection and reporting systems** Reliable systems that regularly tracks and provides feedback on the performance of key processes and outcomes. Large-scale implementation cannot occur or be sustained unless routine data systems are accurate, complete, and timely. Data that tracks key processes and outcomes that are targeted by the intervention need to be shared frequently with frontline staff and system leaders to inform ongoing improvement.	Directly linked with the “infrastructure” theme since the focus was usually on using embedded data systems, including electronic health records, and open data platforms.	*With the rapid growth of patient level datasets based on electronic medical records or pharmacy claims data, the potential for feedback interventions to improve prescribing safety is considerable, and many healthcare systems could deploy similar interventions now.* [54] *Routinely collected, accumulating data in administrative data sets offers a cost-effective opportunity to implement and evaluate antimicrobial stewardship interventions at scale across large populations.* [60] *Since the underlying data are all publicly available, feedback of this kind could be provided by many different interested parties.* [61] *Open data platforms can provide a low-cost route for wide-scale audit and feedback.* [38]
** Learning systems** A mechanism for collecting, vetting, and rapidly sharing change ideas or interventions.	Mainly focused on the benefits of implementation laboratories, clinical networks, or taking a “learning health systems” approach.	*The* [name] *programme effectively represented a nascent ‘implementation laboratory’ embedded within 10 CCGs. It is possible to develop and test incremental ways of improving the delivery of health care that cumulatively both improve patient care and develop the scientific basis of health-care provision. … Embedding trials in an existing network or major improvement initiative facilitates recruitment and helps ensure ‘real-world’ generalisability. We recommend that researchers build collaborations with those responsible for large-scale regional or national improvement to establish implementation laboratories.* [29] *This study demonstrates the benefits of health system–academic collaborations on delivery innovations and the ability to scale nudges when they are codeveloped between clinicians and health systems.* [62] *Clinical networks are increasingly being viewed as a vehicle through which evidence-based care can be embedded into healthcare systems using a collegial approach to agree on and implement a range of strategies within hospitals.* [58]
** Design for sustainability** Plan for the intervention to be sustained.	Covered in the “sustainability” coding Quotes are about the need to consider sustainability and scalability.	*Audit and feedback is a pragmatic, scalable intervention to improve antibiotic use, and when coupled with evaluation systems using administrative databases it could generate sustainable and large reductions in antibiotic use.* [60] *These interventions should be designed to fit into routine primary care practice and policy settings to ensure effectiveness, sustainability, and scalability.* [18] *Although a transient increase in thrombolysis rates was evident during the active phase of implementation support, the negative overall result of the* [name] *trial confirms the recognized challenge of delivering and sustaining health systems change and suggests the need for further implementation research into novel strategies for thrombolysis implementation at scale.* [36]

^a^Definitions summarized from Barker et al. [13]

**Table 4:** Results from inductive analysis for themes related to spread/scale

Theme	Key quotes
**Aligning affordability and scalability:** Intervention studies are typically resource-intensive and high cost, which can be barriers to scaling-up	*Although the added costs of such resource-intensive support* [intensive training, site-visits etc.] *can be maintained during research evaluations, it is challenging to incorporate these costs into a business model that enables sustainable, scalable provision of the service.* [47] *Routinely collected, accumulating data in administrative data sets offers a cost-effective opportunity to implement and evaluate antimicrobial stewardship interventions at scale across large populations.* [60] *A key advantage of automated feedback interventions is that the cost of scaling delivery across entire health systems is much less than for more intensive interventions.* [54]
**Balancing fidelity and scalability:** Maintaining fidelity to the initial study is not always feasible at scale, particularly for complex interventions	*There are questions about whether more complex interventions can be scaled successfully and feasibly, since they are often resource intensive.* [61] *Our intervention, … shows that the favourable results of earlier work could not be replicated. It appeared that large-scale uptake of evidence-based but complex implementation strategies with a minimum of influence of external researchers, but with the stakeholders in healthcare themselves being responsible for the work that comes with integrating this intervention into their own groups, was not feasible.* [44]
**Balancing effect size and scalability:** Scalable interventions may not lead to the same beneficial outcomes as the original trial; however, when delivering interventions at scale, a small effect can still have a large impact	*Improving health system performance by even a small margin has the potential to make a major effect on disease burden if improvements can be delivered at scale.* [25] *These findings suggest that low-intensity, wide-reach CME* [Continuing Medical Education] *programs may be more effective at improving processes but not outcomes of care.* [48] *Although a change of one pill per prescription may be perceived as a modest effect clinically, it reflects a 7 percent decrease (data not shown) during a period of heightened awareness about opioid risks, implementation of multiple other concurrent interventions (for example, the State of California’s opioid prescription drug monitoring program), and a resulting trend toward less prescribing.* [62]

#### Phase of scale-up: what phase of the scale-up process is the trial working at?

For phase 1: set-up, trials discussed how they prepared the groundwork for the trial to scale, including designing materials and training that could be easily scaled. “*The goal-setting and action-planning worksheet was designed to be readily scalable and was delivered with minimal supports*” [63]. Some studies generically mentioned how the trial was “designed for scale”; however, this mainly focused on keeping costs low and some acknowledgment of tailoring for site-specific needs. Not all aspects of the FGFS definitions were addressed, as there was limited mention about how decisions were made about what would be considered “full scale” or how early adopters were brought on board.

In phase 2: develop the scalable unit, the trials mentioned moving beyond initial design to conduct small pilots to inform what would be taken to the next level. A scalable unit is defined as a small administrative unit (e.g., clinical unit, district) that includes key infrastructural components and relationship architecture that are likely to be encountered in the system at full scale [13]. As an example, one trial discussed their aim to “*pilot test the systems consultation strategy in a small set of primary care clinics to see if the strategy demonstrated feasibility, acceptability, and preliminary effectiveness in improving clinician adherence*” [31]. If effective, a follow-up study was planned for a large-scale RCT, followed by a population-level intervention.

Many of the trials that discussed scale frequently were focused on phase 3: test of scale up, as they conducted the trial across multiple sites/settings with the intention of going to full scale. The main focus was on conducting the trials under usual conditions across a large area. The approach taken in one study was mentioned to increase “*confidence in the wider applicability of trial findings as it replicates guideline implementation activities under standard conditions. We paid close attention to ensuring that the evaluated intervention was embedded in real world practice, and the trial itself involved more than 94% of primary care practices in three geographical areas*” [22]. In this phase, testing of infrastructure, as discussed in support systems (infrastructure), was mentioned regularly, particularly regarding the benefits of having the same data systems (i.e., EMRs) used across sites to facilitate scalability, while acknowledging the challenges of adapting to different site needs. Many trials concluded that they should have done more during phase 1 and phase 2.

For phase 4: going to full scale, there was no standardized way to determine what qualified as “full scale”; however, descriptions such as “across all of Australia,” “across the province,” or “on a national scale” were all treated as “full scale.” Trials at this level typically mentioned work from previous phases first, and although the FGFS suggests less emphasis on learning during this phase, as anticipated for a trial, these trials still focused on learning and results.

#### FGFS: adoption mechanisms

Within the adoption mechanisms, determinants include better ideas, leadership, communication, policy, and a culture of urgency and persistence. Included trials mentioned use of more scalable, or “better” ideas before phase 1, as the emphasis was on learning from the literature, and a need for simple ideas or principles that could improve scalability. For example, some studies focused on use of “nudges,” as they aim to be low-cost, innovative behavioral approaches that have potential to be scalable and align well with A&F [26, 62, 64]. There was little mention of leadership or policy, beyond identifying that leaders were involved, or the trial was conducted in a “live policy context,” rather than the impact of leaders or policies. There was no mention of how communication strategies impacted the scale-up process, and when communication was mentioned, it was more about the intervention itself (i.e., an e-mail intervention). The culture of urgency and persistence was mainly mentioned in study introductions, highlighting the need for the intervention, not about the impact of this urgency.

#### FGFS: support systems (infrastructure)

Within support systems (infrastructure), determinants include human capability for scale-up, infrastructure for scale-up, data collection and reporting systems, learning systems, and design for sustainability. Human capability for scale-up focused on implementing the trial in “usual circumstances,” the benefits of needing as little implementation support as possible, and not to be labor intensive. The focus in this determinant was on how to make it feasible for people to engage with the A&F; however, as with the ISF analysis, there was minimal mention about specific skills to enable scalable A&F processes.

Infrastructure for scale-up was the most frequently mentioned determinant, particularly with the emphasis on using existing data structures for audit results, and a standardized way to share feedback. Scaling across sites/settings that have the same systems was seen as a significant facilitator for scaling-up, such as working in systems with the same EMR, or when data was already collected and accessible. However, only embedding the A&F process into the EMR was not enough, and some trials acknowledged they still needed strong design and implementation processes with some adaptation to local settings and processes.

Data collection and reporting systems were directly linked to infrastructure for scale-up, as both focused on using existing data collection and reporting systems, including EMRs and open data reporting systems. This overlap is likely unique to A&F as the need for audit data is the intervention or strategy, while different intervention types would use the data for monitoring and evaluation. Some studies mentioned learning systems, mainly focused on the benefits of implementation laboratories, clinical networks, or taking a learning health systems approach. Design for sustainability is the FGFS domain focused on planning for sustainability, so is covered by the ISF results.

Three new themes were identified:

- Aligning affordability and scalability: keeping costs low was a main way trials planned for future scalability. Studies mentioned how the high cost and high resource use common in these trials were barriers to scale, with some studies mentioning strategies to keep costs down. “*Brief interventions likely need repeating at regular intervals to achieve sustained improvement, balancing affordability and scalability*” [65]. How to align the need for an affordable intervention with the plan for the intervention to be scaled was a frequently mentioned concern. “*Although it was designed with wide reach and scaling up in mind, our budget for Website development and implementation likely exceeded that available… raising concerns about sponsorship of such programs*” [48]. Using existing infrastructure and data reporting systems were key strategies to reduce costs. “*Routinely collected, accumulating data in administrative data sets offers a cost-effective opportunity to implement and evaluate antimicrobial stewardship interventions at scale across large populations*” [60].
- Balancing fidelity and scalability: there were strong concerns about how to maintain fidelity to previous trials while delivering the intervention at scale, particularly for complex interventions. “*Although an all encompassing intervention is likely to achieve impact, complex interventions can be impractical to scale up*” [66]. Some trials selected key elements of a previous trial to scale, while others tried to maintain fidelity, yet typically indicated more preparation work was needed.
- Balancing effect size and scalability: although studies had concerns about smaller effect sizes than anticipated based on a pilot study, some trials acknowledged how this small effect at a large scale led to greater impact overall. “*Although this is a small change for an individual prescriber, our study demonstrates how this can lead to large impacts on antibiotic use over a broad jurisdiction*” [60]. The recognition of this impact potential was a driving force for trials that aimed to be implemented at scale. “*Scalable and effective systems that require minimal support to implement could make major improvements in primary healthcare system performance and health outcomes globally*” [25].

## Discussion

A&F trials should plan for sustainability, spread, and scale so that if the trial is effective, the intended benefit can continue and benefit a wider audience, which also reduces research waste and increases trust from the community [2–8]. Sustainability periods ranged from 2 to 24 months, with 12 months used most frequently. Although 78% of included studies mentioned a keyword related to sustainability, only 38% mentioned it frequently, and this was usually in vague statements in the discussion with suggestions for how it could be sustained, if effective, not how it was sustained. Similar findings applied for spread and scale. This lack of experience, specificity, and detail makes it difficult to recommend concrete strategies related to barriers and facilitators to A&F sustainability, since we know sustainability planning benefits from careful consideration of sustainability determinants [7]. Mapping to the ISF provided some insight into the broader domains and determinants that shape sustainability of A&F as tested in trials, which are vital for planning for their sustainability. Planning for scale mainly focused on keeping costs down and using existing infrastructure, without acknowledging the role of other mechanisms, such as policy, leadership, and communication, that support scale.

Twelve months was the most frequent sustainability duration reported, but total study durations and sustainability periods were not clearly reported in many studies. As different terminology was used across studies, with many not explicitly calling it a sustainability period, some of these time periods were included when it may not have been considered by the trial authors to be measuring sustainability. There is currently no recommended time for claiming an intervention is sustained; however, 12 months may not be long enough to truly understand whether or not an intervention, implementation strategy, and/or impact are sustained. Authors are encouraged to report clearer sustainability durations, publish follow-up studies, and indicate if the intervention, including implementation strategies, continued or not during that time.

The ISF determinants provided a useful structure to explore what may impact sustainability of A&F-based interventions, although it was difficult to directly connect ISF determinants to A&F, rather than other components of the intervention (education, champions etc.). Using the ISF is recommended to design suitable and appropriate sustainability strategies for future A&F trials, alongside tools such as the Expert Recommendations for Implementing Change (ERIC) sustainability glossary [67], which may be useful for determining specific strategies when planning for A&F sustainability. Our difficulty differentiating between implementation and sustainability characteristics is common within sustainability research [4, 7] and demonstrates the interconnected nature of these characteristics. This interconnectedness may also reiterate the need to consider and plan for sustainability early, during initial implementation [8]. The FGFS was useful to categorize phases of scale-up and for highlighting what was, and was not, discussed within trial descriptions. The FGFS may be a useful guide to plan ongoing scale-up of A&F processes, particularly as an overarching guide to help avoid the common mention of the need for more planning when the effect was not seen when delivered at scale.

As limited work has been conducted regarding sustainability of A&F, this qualitative review was important to conduct before asking questions about sustained effectiveness of A&F. With confusion around the definition and timeline of sustainability (range from 2 to 24 months), lack of clarity on whether the intervention was continued during the sustainability period, and generally inconsistent reporting, clear criteria, informed by this review, will be needed going forward when exploring sustained effectiveness of A&F trials. Trials will likely need to report results for at least three time points (baseline, end of intervention, and post-intervention), have a minimum amount of time that qualifies as “sustained,” and a clear differentiation between trials that continued the intervention and implementation strategies, including A&F, after the intervention phase and those that did not. Further exploration of scale will also need more consistency regarding the scalability phase of the trial, particularly what is meant by “full scale.” Improved reporting of intervention timelines and increased descriptions of how sustainability and scalability were planned (in the original or subsequent publications) will help increase our understanding of this impactful topic.

### Limitations

We limited eligibility to more recent trials given the more recent focus in the literature on sustainability, spread, and scale, but recognize that in doing so, some insights from older studies would be missed.

Results are based on A&F trials designed to look at effectiveness within clear time limits, so the lack of detail regarding sustainability and spread/scale planning was unsurprising. We mitigated this limitation through the forward citation search. As included trials often used multiple intervention components and implementation strategies, not limited to A&F, it is not possible to attribute results solely to A&F. Although our initial inclusion criteria based on keywords aimed to be as inclusive as possible, some studies were excluded due to lack of use of specific words. For example, one study always used “12 months” to refer to continuation of the trial and was excluded [68]. As many studies were cluster trials that may need multiple sites, these trials do not necessarily reflect spread/scale; however, given the focus on keywords regarding spread/scale, valuable information was learned about sustainability, spread, and scale from trials conducted at multiple sites. Cluster trials were also conducted at the level of sub-team, ward, or even clinician. With the limited focus on sustainability within these trials, we chose to focus on all mentions of the topic rather than differentiating between sustainability of the intervention post-trial and sustainability of the effect of the intervention on behavior change, or outcomes. As more focus is placed on how to sustain A&F processes and subsequent behavior change, further distinction should be made between these sustainability indicators and time periods.

We also acknowledge that these studies were not necessarily solely or explicitly designed to study sustainability, spread, or scale, and future work could focus on studies with this explicit focus.

Our initial aim was to extract text directly to the ISF and FGFS; however, there was a large discrepancy between reviewers during the first pilot due an inability to distinguish between text explaining the initial implementation versus information specific to sustainability/spread/scale. For this reason, the broader strategy for text extraction was used as it had more consistent extraction during the second pilot. This change meant that potentially relevant text for the frameworks may not have been extracted if it was not directly referring to sustainability, spread, or scale. This method may explain why limited information was found for factors of the ISF and adoption mechanisms of the FGFS; however, the general lack of detail regarding these planning strategies indicates that a different extraction process would likely have led to the same results.

## Conclusion

A&F trials should plan for sustainability, spread, and scale so if effective, the benefit can continue and impact a wider audience. Many studies lacked detail on if or how they planned for any aspect of the intervention, including A&F, to be continued. Scalability planning must go beyond keeping costs low and using existing infrastructure, to considering other strategies that support scalability. Future research should explore if the effect of an A&F trial is continued, for how long, and whether this is with or without continuation of the A&F process. Careful planning for sustainability, spread, and scale is needed to ensure that the changes can have a positive, sustainable, impact for a wide audience across different contexts.

### Supplementary Information

**Additional file 1: Appendix 1. **Full list of keywords used for sustainability, spread, and scale.

**Additional file 2: Appendix 2. **Methods for grouping studies.

**Additional file 3: Appendix 3.** Codebook.

## Data Availability

The datasets used and/or analyzed during the current study are available from the corresponding author on reasonable request.

## Abbreviations

A&F: Audit and feedback
FGFS: Framework for Going to Full Scale
ISF: Integrated Sustainability Framework
RCT: Randomized control trial
