# Supplementary material for: Sustainability, spread, and scale in trials using audit and feedback: a theory-informed, secondary analysis of a systematic review
Source: Implement Sci. 2023 Oct 26;18:54. doi: 10.1186/s13012-023-01312-0 (PMC10604689; doi:10.1186/s13012-023-01312-0)
Supplement: Supplementary file 1 — Additional file 1: Appendix 1. Full list of keywords used for sustainability, spread, and scale. [file 13012_2023_1312_MOESM1_ESM.pdf]

Appendix 1: Full list of keywords used for sustainability, spread and scale.

Sustainability

sustain*
- sustaining
- sustainable
- sustainably
- sustained
- sustainability
- other sustain*

maintain*
- maintenance
- maintained
- maintaining
- other maintain*

normal*
- normali#ation
- normali#e
- other normal*

institutional*
- intitutionali#e
- other institutional*

integrat*
- integrating
- integration
- integrated
- other integrate*

embed*
- embedding
- embedded
- other embed*

durab*
- durability
- durable
- other durability*

long*
- longitudinal
- other longitudinal*
- long-term*
- longer-term*
- other long-term*

routin*
- routinized
- routinization
- other routine

standard*
- standardi#e
- standardi#ation
- standardi#ed
- other standard

Spread/Scale

Scal*
- scales
- scaling
- scaled
- scal*-up
- up-scale
- up-scaling
- upscaled
- scaling-out
- scalable
- scalability
- at scale
- other scal*

spread
- spreading
- spreads
- widespread
- other spread*

roll
- roll out
- rolling out
- rolled out
- other roll*

reach
- other reach

generali#e*
- generali#ed
- generali#able
- generali#ability
- generali#es
- other generali#e
