# Supplementary material for: Sustainability, spread, and scale in trials using audit and feedback: a theory-informed, secondary analysis of a systematic review
Source: Implement Sci. 2023 Oct 26;18:54. doi: 10.1186/s13012-023-01312-0 (PMC10604689; doi:10.1186/s13012-023-01312-0)
Supplement: Supplementary file 2 — Additional file 2: Appendix 2. Methods for grouping studies. [file 13012_2023_1312_MOESM2_ESM.pdf]

Appendix 2: Methods for grouping studies.

Due to the variation in the amount of focus each study placed on sustainability and spread/scale, studies were grouped prior to analysis. Groupings were decided separately for sustainability and spread/scale based on a series of tests using 15 studies each that were deemed to have a significant focus on sustainability or spread/scale. Tests were based on how many of the 15 studies would be included if the grouping was based on three or more keywords, text in two or more locations, and, for sustainability only, if the author claimed to be measuring sustainability. For sustainability, the author claim was deemed inappropriate as some studies that frequently discussed sustainability did not claim to be measuring sustainability; and thus, not all of the 15 tester studies would be included.

For word frequencies, all 15 tester studies would be included if three or more keywords were used for sustainability, yet only 6/15 would be included for spread/scale. For number of text locations (abstract, summary box, introduction, methods, results, discussion, conclusion, Table/Figure, and other), all 15 studies were included when two or more, and three or more locations were used as criteria for sustainability. All 15 studies were included for two or more for spread/scale, yet only 10/15 were included for three or more locations. The final decision for sustainability was to include all studies that had text extracted from three or more locations to undergo comprehensive analysis (termed "Frequent Sustainability"). All other studies that included at least one sustainability key word underwent content analysis only (termed "Occasional Sustainability"). For spread/scale, all studies that had text extracted from two or more locations underwent comprehensive analysis (termed "Frequent Spread/Scale"); all other studies that included at least one spread/scale key word underwent content analysis only (termed "Occasional Sustainability"). As the key word "generalizabl*" was deemed to have a relevant but unique meaning, studies that were only included due to their inclusion of this keyword were grouped separately.

Qualitative analysis was conducted by two researchers (CL and ZL) using NVivo 12. Four separate analyses were conducted including:

1) *Frequent Sustainability:* deductive analysis to the Integrated Sustainability Framework + inductive analysis to pre-specified codes
2) *Occasional Sustainability:* inductive analysis (same codes as for *Frequent Sustainability*)
3) *Frequent Spread/Scale:* deductive analysis to the Framework for Going to Full Scale + inductive analysis to pre-specified codes
4) *Occasional Spread/Scale:* inductive analysis (same codes as for *Frequent Spread/Scale*)
