# Supplementary material for: Sustainability, spread, and scale in trials using audit and feedback: a theory-informed, secondary analysis of a systematic review
Source: Implement Sci. 2023 Oct 26;18:54. doi: 10.1186/s13012-023-01312-0 (PMC10604689; doi:10.1186/s13012-023-01312-0)
Supplement: Supplementary file 3 — Additional file 3: Appendix 3. Codebook. [file 13012_2023_1312_MOESM3_ESM.pdf]

Audit & Feedback Sustainability Sub-Review Codebook

SUSTAINABILITY

INDUCTIVE ANALYSIS

(Full Analysis + Content Analysis)

**Domain**	**Definition**	**Example**
Few studies consider sustainability (GENERIC)	All text that mentions that few studies consider sustainability of A&F interventions.<br>(Typically in the introduction, when discussing that other studies did not consider sustainability)	*"So, for example, interventions designed to encourage the routine use of WHO disease-specific guidelines in rural pediatric hospitals have not been tested."*<br>*"Much previous research has focused on pharmacist led interventions, although the effect of such interventions is variable and sometimes negative, at least partly owing to lack of integration with existing primary medical care."*
Further study of sustainability is needed<br>(FOR THIS TRIAL)	All text that mentions the need to consider/study sustainability for this trial but does not say how.<br>(Text that mentions need for sustainability studies more generally can go in "Few studies consider sustainability)	*"More studies are needed to determine whether the effect observed for the intervention is maintained in time, although such studies are difficult to conduct in everyday practice."*<br>*"The sustainability of the impact of this type of intervention needs further study since there are discrepant data on the long-lasting effects of these types of interventions."*<br>*"It was also beyond the scope of our study to examine whether guideline improvements continued to improve or were sustained beyond 1 year postintervention."*

Domain	Definition	Example
Plans for measuring sustainability<br><br>(FOR THIS TRIAL)	All text that includes the aim to measure sustainability. Details on how this is measured could be included in the ISF analysis.<br><br>Can include use of theories to design the intervention (i.e. NPT)<br><br>(Typically in Methods. Ideally most methods would be included in the ISF, but if the methods are generic they can fit here).	*"Guided by normalisation process theory, the research team worked with each clinic to tailor the intervention to the clinic and embed it into routine practice."*
Potential for sustainability<br><br>(FOR THIS TRIAL)	All text that mentions the potential for sustainability but does not say how or provide any details.	*"Our analysis was limited to prescribing changes that occurred in the intervention group in the first year after the portrait. It is conceivable that the portrait produced an impact on prescribing that lasted more than 1 year, in which case our estimates would have underestimated long-term benefits to patients and cost savings."*<br><br>*"Longer follow-up may show a greater decline in antibiotic prescription rates, or separation in the relative effectiveness of the PDS and CDS strategies. Conversely, longer follow-up could also show a regression of the intervention effects."*
Results – sustainability was/was not achieved<br><br>(FOR THIS TRIAL)	All text that mentions if the intervention was sustained or not.<br><br>(Typically, in Results and/or Conclusions for generic statements that don't fit into the ISF).	*"These findings do not support the routine use of our intervention among unselected institutions."*

INTEGRATED SUSTAINABILITY FRAMEWORK

(Full Analysis ONLY)

Domain	Factors covered + Example	Factor definition	Questions to Consider
**Outer / Policy contextual factors**	• Policy and legislation<br>• Sociopolitical context<br>• Funding environment<br>• Leadership<br>• Values, priorities, needs<br>• Community ownership<br><br>*"The results have implications for both the redesign of the intervention in an effort to achieve greater and more sustainable change and the development of higher-level policy for improvement in stroke thrombolysis implementation, addressing issues such as expert workforce capacity building, healthcare management accountability to benchmarks, and incentives for achieving benchmark performance in IVT."*<br><br>*"Thus, strategies that target compliance determinants both at policy, organizational, leadership,*	**Sociopolitical context:** The external landscape, including existing policies and regulations, guidelines, and mandates that pose implications on the sustainment of evidence-based interventions (EBIs). This may also include sociocontextual norms or policies that are discriminatory or stigmatising. (Focus of the impact of these factors on continuing the intervention after the trial, not in initial set-up.)<br><br>**Funding environment and availability:** The funding landscape, including nature, stability, scope, diversity, and length of the funding environment after the trial ends.<br><br>**External partnerships and leadership/environmental support:** Receiving external support through networks and partnerships (e.g., through engagement or resource exchange with academic and health organisations and community partners), support, commitment, and involvement from national leadership.<br><br>**Values, needs, priorities:** The extent to which an EBI or topic is regarded as a national priority	What **policies, regulations, and social norms** are in place that may have implications for sustainability?<br><br>What's the **broader funding environment** like and are there external funds that could help sustain the EBI?<br><br>Are there **external partnerships (with government agencies, healthcare systems, community-based organizations)** that can help bring resources, support, and commitment to sustain the EBI?<br><br>How does EBI align with **national, state, local priorities**?

Domain	Factors covered + Example	Factor definition	Questions to Consider
	team, and individual levels [6] may probably be able to achieve and sustain compliance improvements and NI reductions which exceed those in the HELPING HANDS- and the PSYGIENE-trial."	or fits with national, state, or local organisational priorities, needs, and values.	
**Inner / Organizational contextual factors**	• Funding/resources<br>• Leadership/support<br>• Climate/culture<br>• Staffing turnover<br>• Structural characteristics<br>• Capacity<br>• Champion<br>• Polices (alignment)<br>• Mission<br><br>*"The problems on the fidelity of the feedback and with the uptake of the intervention could best be handled by assuring that a strong leader picks up the group and lead them forward."*<br><br>*"Thus, strategies that target compliance determinants both at policy, organizational, leadership, team, and individual levels [6] may*	**Programme champions:** Individuals who have strong influences on the behaviours, attitudes, and norms of their colleagues/peers and promote the ongoing delivery of EBIs. This does not include the trial researchers, unless they are also part of the organization.<br><br>**Organisational leadership/support:** Support from those within the organisation who have formal responsibility for leading, organising, and overseeing the programme. This does not include the trial researchers, unless they are also part of the organization.<br><br>**Organisational readiness/resources:** The level of resources and support internally to facilitate the ongoing delivery of a programme (e.g., space, money, funding, time).<br><br>**Organisational stability:** Staff attrition and turnover of space, organisation, staffing, or leadership during and after the trial has ended.	Are there **program champions** (community and organizational) who can help influence sustained delivery of the EBI?<br><br>Does the EBI have **support from organizational leadership**?<br><br>Within the organization, is there **organizational infrastructure** (time, financial resources, space) to support the EBI? How 'ready' is the organization?<br><br>How are **stakeholders continually engaged** related to EBI delivery?

Domain	Factors covered + Example	Factor definition	Questions to Consider
	probably be able to achieve and sustain compliance improvements and NI reductions which exceed those in the HELPING HANDS- and the PSYGIENE-trial."		
**Implementation Processes**	• Partnership/engagement<br>• Training/support/ supervision<br>• Fidelity<br>• Adaptation<br>• Planning<br>• Team/board functioning<br>• Programme evaluation/data<br>• Communication<br>• Technical assistance<br>• Capacity building<br><br>*"We provided a 2-h interactive training session (integrated within routine training procedures to improve replicability) for doctors within the first month following the implementation of the intervention."*	**Partnership/engagement:** Processes to directly and actively engage with key stakeholders (e.g., community board, role modelling, and networking).<br><br>**Training/supervision/support:** Processes related to implementation strategies (e.g., formal education or training, formal supervision processes, or other forms of support).<br><br>**Programme evaluation/data:** Collection of data and assessment or feedback to inform programme planning and decisions outside of/after the trial ends.<br><br>**Adaptation:** Processes in place to actively and systematically guide adaptation after completion of the trial.<br><br>**Communications and strategic planning:** Processes explicitly related to or that guide the sustainment of a programme over time, e.g., through grant-writing, activities and	Are there processes in place to support the **recruitment and retention** of staff involved with EBI delivery?<br><br>Are there **supervision and training** processes in place to support EBI delivery among staff over time?<br><br>Are there processes in place or that could be added to **track or monitor data** on health impact of EBI or its delivery?<br><br>Is there **strategic planning** about sustaining the EBI (e.g. grant writing, communications)?

Domain	Factors covered + Example	Factor definition	Questions to Consider
		engagement regarding sustainment, or marketing/communication plan focused on promoting the sustainment of an EBI.	
**Implementer and population characteristics**	• Provider/implementer characteristics<br>• Implementation skills/expertise<br>• Implementer attitudes<br>• Implementer motivation<br>• Population characteristics<br><br>*"In the present research, this suggests that interventions that are embedded into the consultation environment and become active during the flow of care are more likely to be successful. Social cognitive theory also proposes that the strength of an individual's belief in his/her own ability to reach goals (i.e. their self-efficacy) functions as a key determinant of motivation for a specific behaviour."*	**Provider/Implementer characteristics:** Implementer role self-efficacy, role clarity, commitment, and attitude. This does not include research team members unless they are also in the organization receiving the trial.<br><br>**Provider/Implementer benefits and stressors:** Implementer benefits and stressors in role (including if paid or a volunteer). This does not include research team members unless they are also in the organization receiving the trial.<br><br>**Provider/Implementer skills/expertise:** Prior knowledge, training, and motivation of the implementer. This does not include research team members unless they are also in the organization receiving the trial.<br><br>**Population characteristics:** Trust and medical mistrust, literacy, socioeconomic status, race/ethnicity, and experiences of stigma or discrimination among the target population.	Do the implementers have the **self-efficacy** to deliver the EBI over time?<br><br>What are some of the **benefits and challenges** that implementers might experience in delivering the program over time?<br><br>What are the **attitudes** of the implementers towards the EBI?<br><br>What **characteristics or experiences of the population** served might impede sustainability (e.g. stigma, mistrust, literacy, poverty, experiences of discrimination)?
	• Adaptability<br>• Fit with population and context	**Adaptability of EBI/fidelity:** Degree to which an EBI can be tailored or refined to fit new	How **adaptable** is the EBI?

Domain	Factors covered + Example	Factor definition	Questions to Consider
**Characteristics of the intervention**	• Benefits/need<br>• Burden/complexity<br>• Trialability<br>• Cost<br><br>*"However, such adaptation would need to be complemented by careful consideration of how systems can be made ready to support implementation of new practices and improved quality of care. We would suggest this includes due attention to influencing the institutional culture and context of rural hospitals although willingness to invest in more integrated approaches often seems lacking."*<br><br>*"JITIF is effective in controlling antibiotics prescription at least in the short term and may provide a low-cost and sustainable solution to the widespread excessive use of antibiotics in rural China."*	settings or population needs, e.g., original guidelines or evidence vs. newer guidelines or evidence.<br><br>**Fit with context/population/organisation:** Fit of an EBI within a context, populations, and organisations as well as the perceived trust and medical mistrust of an EBI or source of evidence.<br><br>**Perceived benefits**: Perceived impact, evidence, cost, or relative advantage of an EBI. This includes results of the trial (i.e., was the trail effective or not).<br><br>**Perceived need**: Perceived need in the community or setting for an EBI or the topic it addresses.	How **costly** is the EBI? Is there a return on investment?<br><br>How well does the **EBI 'fit'** within the organizational context?<br><br>Does the EBI continue to **address a priority or need** in the community?

SPREAD/SCALE

INDUCTIVE ANALYSIS

(Full Analysis + Content Analysis)

Domain	Definition	Example
Few studies consider spread/scale (GENERIC)	All text that mentions that few studies consider spread/scale of A&F interventions.	*"There are questions about whether more complex interventions can be scaled successfully and feasibly, since they are often resource intensive."*<br><br>*"Previous large-scale implementations of such interventions have focused on a single practice and have not been randomized, thus limiting causal inferences and generalizabilty."* [Also code in "Generalizability"]
Further study of spread/scale is needed<br><br>(FOR THIS TRIAL)	All text that mentions the need to consider/study how to bring the intervention to another setting/wider use but does not say how.	*"Although these findings seem to suggest that efforts to implement and scale up improved secondary pediatric health care will need to include more than the production and dissemination of printed materials, further research including trials or evaluation of test programs are necessary before wide spread adoption of any multifaceted approach (which will need to be tailored to local conditions and available resources) can be contemplated."*<br><br>*"A larger scale, multicenter trial of this type of intervention directed at attending staff level physicians and/or physician extenders is warranted to determine whether this approach will be successful in other practice environments."*<br><br>*"Although a transient increase in thrombolysis rates was evident during the active phase of implementation support, the negative overall result of the TIPS trial confirms the recognized challenge of delivering and sustaining health systems change and suggests the need for further implementation research into novel strategies for thrombolysis implementation at scale."*

Domain	Definition	Example
**Potential** for spread/scale<br>(FOR THIS TRIAL)	All text that mentions the *potential* for the intervention to be used elsewhere (spread/scale).	*"This intervention consists of widely accessible components and has been tested in a real-world setting, and is therefore well positioned for use at scale."*<br>*"The sustainability of the intervention was reflected by continued first-line prescribing at 5 months after the intervention. The success of the SIMPle study has garnered the interest of the Irish College of General Practice, and a national rollout is planned."*
Intervention **is** spread/scaled or delivered at "large scale"<br>(FOR THIS TRIAL)	All text that mentions that the intervention is being applied 'at scale', nationally, or across a wide region/setting (full health system etc.) etc.	*"Providing new tools does not however necessarily change practice. A large scale-up in the deployment of malaria RDTs by national malaria control programmes from less than 200,000 in 2005 to more than 108 million in 2012 has been undertaken."*<br>*"The present large-scale randomised trial investigates the effect of a hands-on training course for leading colonoscopists at screening centres to improve their adenoma finding skills."*
Generalizability	All mention of generalizability to be coded here.	*"Further research is needed to understand the generalizability of these findings."*

IHI Framework for Going to Full Scale

(Full Analysis ONLY)

Developed from here: https://implementationscience.biomedcentral.com/articles/10.1186/s13012-016-0374-x

NOTE: The text in the paper and Figure DO NOT MATCH! I've used the text, not the figure, as the text provides the detail.

Domain	Definition	Example
Phase of Scale-Up: What phase of the scale-up process is the trial working at?		
**Phase 1: Set Up**	- Prepares the ground for introduction and testing of the intervention that will be taken to full scale<br>- establishes an entry point for the planned intervention into the existing health system.<br>- includes a clear articulation of what needs to be scaled up and defines the ambition for "full scale."<br>- initial test sites, early adopters, and *potential* "champions" of the intervention are identified.	*"The goal-setting and action-planning worksheet was designed to be readily scalable and was delivered with minimal supports."*<br>*"Users from all pilot sites reported that training in the form of education and educational resources would be required for a national rollout of the ART."*
**Phase 2: Develop the Scalable Unit**	- An early test and demonstration phase<br>*Scalable unit:* typically, a small administrative unit (e.g., sub-district/district or clinical ward/division) that includes key infrastructural components and relationship architecture that are likely to be encountered in the system at full scale.<br>- If the ambition of scale is large (e.g., county, province, health system), a scalable unit could comprise multiple levels of care and the communities that are served by a	*"Nudges to physicians offer an effective, low-cost, and scalable approach to increase use of automated patient dashboards to improve guideline-concordant prescribing behaviors."*

Domain	Definition	Example
	large health system, or a divisional unit of care in a hospital setting or large clinic system.	
**Phase 3: Test of Scale-up**	- Testing the set of interventions to be taken to scale<br>- spreads the intervention to a variety of settings that are likely to represent contexts that will be encountered at full scale;<br>- The underlying theory of change and the change package from a successful early demonstration need to be tested in a broader range of settings before going to full scale.<br>- Test necessary infrastructure (e.g., data systems and supply chain) required to support full-scale implementation and build the human capacity and capability (e.g., leadership, managerial, and frontline capacity needed to support the method being used to scale up).<br>- important opportunity to build the belief and will of leaders and frontline staff to support the changes.<br>DETAILS ABOUT THE INFRASTRUCTURE IS BELOW, NOT HERE – THIS IS JUST ABOUT *IF* THE STUDY IS *TESTING* THIS INFRASTRUCTURE	*"To get a geographic spread of the intervention and control, the nursing homes were also divided into three geographical areas."*
**Phase 4: Go to Full Scale**	Unfolds rapidly to enable a larger number of sites to adopt and/or replicate the intervention	*"The intervention was designed for scale-up through its integration within the rural health system in China,*

Domain	Definition	Example
	- a well-tested set of interventions, supported by a reliable data feedback system, is adopted by frontline staff on a larger scale.<br><br>- The focus is on rapid uptake of the intervention through replication.<br><br>- While some adaptation of the intervention to local environments will always be required, there is *less emphasis on new learning* in this phase.	*and could be adapted to other similar settings in LMICs facing the problem of antibiotic overuse."*
Adoption Mechanisms		
Better Ideas	- Ideas that are designed for scalability<br><br>- evident superiority of the intervention<br><br>- simplicity<br><br>- alignment with the culture of the new implementers	*"The goal-setting and action-planning worksheet was designed to be readily scalable and was delivered with minimal supports."*<br><br>*"The study will be one of the first randomised controlled trials to test the effectiveness of clinical networks to lead changes in clinical practice in hospitals treating patients with high-risk cancer. It will additionally provide direction regarding implementation strategies that can be effectively employed to encourage widespread adoption of clinical practice guidelines."*
Leadership	- the capacity for leading large-scale change needs to be developed as part of the scale-up process.	*"Before beginning the QI project, 1 author (J.V.T.) piloted the training in 1 clinic. He refined the approach on the basis of physicians' feedback and then prepared physician training leaders to deliver it*

Domain	Definition	Example
	- Leaders can be coached to understand the difference between simply raising awareness of a new practice and what it takes to lead and ensure its broad adoption.	*through an inperson "train the trainer" session. Training leaders were compensated for time spent traveling to and facilitating the training sessions."*
Communication	- communicating the value of the intervention to both leadership and the implementers (frontline staff).	*"System, structural, and organisational support for system-wide changes to enable implementation strategies to be rolled out and scaled up (e.g., legislation, resources, mechanisms for communication and collaboration between health sectors)"*
Policy	- The identification and/or development of regulatory or administrative policies<br>- Policy can have a supportive or disruptive effect	*"These interventions should be designed to fit into routine primary care practice and policy settings to ensure effectiveness, sustainability, and scalability."*
Culture of Urgency and Persistence	- Consideration of why others would want to join the effort and whether there is a glaring gap in performance or an urgent need<br>- Checking the amount of will and energy needed to stay the course in bringing interventions to—and achieving results at—full scale.<br>- Levels of will and energy may fluctuate over time	*"As research continues to yield evidence-based strategies for improving the delivery of HPV vaccine and other preventive services, health care systems urgently need support in scaling up these strategies efficiently and without reliance on external funding."*
Support Systems (Infrastructure)		

**Domain**	**Definition**	**Example**
Human *Capability* for scale-up	- scale-up will require team leaders who can use change management approaches to guide and mentor teams at the front line and improvement specialists who can lead and design QI-based programs for those who need additional training.<br><br>- The project needs be able to communicate quantitative results and the underlying stories of success and challenge.<br><br>- Data managers need training in analytic and reporting capabilities that are best suited to QI methods (e.g., run charts and statistical process control).	*"Users from all pilot sites reported that training in the form of education and educational resources would be required for a national rollout of the ART."*
Infrastructure for scale-up	- Common structural considerations include:<br>○ additional tools (e.g., checklists, data capture systems),<br><br>○ communication systems (e.g., materials and messages, mentoring relationships, structured programs),<br><br>- key personnel (e.g., data capturers, quality improvement mentors)	*"Pharmacists were supported by a combination of intensive direct training, audit and feedback of their performance on the quality of medication-use measures, regular on-site visits and telephone calls to support and sustain the implementation of the service throughout the evaluation. Although the added costs of such resource-intensive support can be maintained during research evaluations, it is challenging to incorporate these costs into a business model that enables sustainable, scalable provision of the service."*
Data collection and reporting systems	- a system for regularly delivering the feedback as part of A&F	*"However, those studies either used labor-intensive manual chart audit and in-person feedback from peers or combined SCF with other resource-intensive interventions such as provider education. With increased use of EMR based dashboards by healthcare*

Domain	Definition	Example
	- reliable systems that track and provide feedback on the performance of key processes and outcomes<br><br>- large-scale implementation cannot occur or be sustained unless routine data systems are accurate, complete, and timely.<br><br>- Data that tracks key processes and outcomes that are targeted by the intervention need to be shared frequently with frontline staff and system leaders to inform ongoing improvement.	*systems, automated SCF alone represents an easily scalable intervention to influence frontline providers' treatment patterns."*<br><br>*"However, a key advantage of automated feedback interventions is that the cost of scaling delivery across entire health systems is much less than for more intensive interventions."*
Learning Systems	- a mechanism for collecting, vetting, and rapidly sharing change ideas or interventions.<br><br>- Any mention of a "Learning Health System"	*"In primary care, multiple priorities and system pressures make closing the gap between evidence and practice challenging. Most implementation studies focus on single conditions, limiting generalisability. We compared an adaptable implementation package against an implementation control and assessed effects on adherence to four different evidence-based quality indicators."*
Design for Sustainability	- ANY STATEMENT THAT MENTIONS SUSTAIN + SPREAD/SCALE<br><br>- Plan for the intervention to be sustained	*"The sustainability of the intervention was reflected by continued first-line prescribing at 5 months after the intervention. The success of the SIMPle study has garnered the interest of the Irish College of General Practice, and a national rollout is planned."*
